# Association Between Levodopa with Inotrope Prescription and Mechanical Ventilation Dependence in People with Parkinson’s Disease upon Septic Shock

**DOI:** 10.3390/jcm14030748

**Published:** 2025-01-24

**Authors:** Yun-Yung Cheng, Chien-Tai Hong, Li-Ying Chen, Yu-Hsuan Shao, Wei-Ting Chiu, Lung Chan

**Affiliations:** 1 Department of Neurology, Shuang Ho Hospital, Taipei Medical University, New Taipei City 23561, Taiwan; 20289@s.tmu.edu.tw (Y.-Y.C.); ct.hong@tmu.edu.tw (C.-T.H.); 2 Department of Neurology, School of Medicine, College of Medicine, Taipei Medical University, Taipei 11031, Taiwan; 3 Health Data Analytics and Statistics Center, Office of Data Science, Taipei Medical University, New Taipei City 23561, Taiwan; lychen@tmu.edu.tw; 4 Graduate Institute of Biomedical Informatics, Taipei Medical University, Taipei 11031, Taiwan; jonishao@tmu.edu.tw

**Keywords:** Parkinson’s disease, septic shock, levodopa, inotropes, mechanical ventilation

## Abstract

**Background/Objectives**: People with Parkinson’s disease (PwP) face high risks of bacterial infections and septic shock, often requiring inotrope treatment and mechanical ventilation. The impact of levodopa on these critical care interventions remains unclear. **Methods**: This retrospective cohort study analyzed data from the Taipei Medical University Clinical Research Database to explore the relationship between levodopa prescription, inotrope treatment duration, and mechanical ventilation dependence in PwP who experienced septic shock. PwP aged 45 years and older who required intensive care for septic shock were categorized into levodopa and non-levodopa groups. **Results**: Outcomes included inotrope treatment duration, mechanical ventilation dependence, and 3-month mortality. Among 243 PwP (116 without levodopa, 127 with levodopa), no significant difference was observed in the duration of mechanical ventilation dependence. However, levodopa prescription was associated with a significantly extended inotrope treatment duration (estimate: 3.43 days, *p* = 0.027). Additionally, a nonsignificant trend was identified between levodopa prescription and reduced 3-month mortality (adjusted hazard ratio = 0.67, *p* = 0.068). **Conclusions**: These findings highlight the complex interplay between Parkinson’s disease management and critical care interventions during septic shock. While levodopa may prolong inotrope use, its potential to influence survival outcomes warrants further investigation.

## 1. Introduction

Parkinson’s disease (PD) is the second most common neurodegenerative disorder, with aging being a significant risk factor. The mean age of onset is between 55 and 65 years, and most people with PD (PwP) are elderly. PwP are at substantially higher risk of hospital admission compared to the general population, with motor complications, psychosis, general medical conditions, and combined motor and psychiatric issues being the primary reasons [1]. For emergency admissions, bacterial infections such as urinary tract infections and pneumonia are particularly prevalent as leading causes [2], second only to falls. These infections surpass stroke, myocardial infarction, and psychosis in frequency and pose significant risks due to the potential progression to septic shock, a severe and life-threatening condition. Beyond the acute consequences of septic shock, the severe systemic inflammation triggered by sepsis may further exacerbate neurodegeneration. Inflammatory conditions have been found to be associated with clinical phenotypes and the progression of PD and other degenerative movement disorders [3,4,5].

The pathological hallmark of PD is the degeneration of dopaminergic neurons in the substantia nigra of the midbrain, along with the accumulation of α-synuclein-enriched Lewy bodies. However, PD pathology extends beyond the midbrain, with degeneration in the pons and medulla often preceding midbrain involvement. This brainstem pathology can significantly impair respiratory and autonomic functions [6]. Ventilatory dysfunction, including dyspnea, may emerge even in the early stages of PD, stemming from impaired brainstem ventilatory control. This dysfunction can present as a premotor manifestation of the disease. PwP often struggle more with repetitive motor tasks than with single actions due to bradykinesia and skeletal muscle rigidity [7,8]. Respiratory complications in PwP include upper airway obstruction and chest wall restriction, both of which may respond to levodopa. However, levodopa-induced respiratory dyskinesia, which can exacerbate respiratory difficulties, complicates treatment. Consequently, PwP often experience impaired cough reflexes, dysphagia, and reduced mobility, all of which predispose them to aspiration pneumonia and infections [9]. In advanced stages, severe infections can result in respiratory failure, frequently necessitating mechanical ventilation.

In addition to motor symptoms, autonomic dysfunction is a significant non-motor feature of PD, encompassing orthostatic hypotension, cardiac arrhythmias, constipation, excessive sweating, and sexual dysfunction. Dysautonomia compromises the cardiovascular system’s ability to regulate responses to infections, further increasing the risk of severe infections. Sepsis is a leading cause of hospital admissions among older adults, regardless of PD status [10]. In PwP, septic shock often requires inotropic support to maintain blood pressure and organ perfusion, given the cardiovascular system’s reduced capacity to combat infection.

Administering levodopa to PwP in septic shock presents a therapeutic dilemma. While levodopa is the cornerstone of PD management [11,12], it can induce hypotension, potentially compromising hemodynamic stability in septic shock. Furthermore, levodopa has been shown to produce dose-dependent hypertension and tachycardia when microinjected into the rostral ventrolateral medulla in animal models [13]. These effects may necessitate prolonged inotropic support, extending intensive care unit (ICU) stays, increasing the risk of nosocomial infections, and delaying hospital discharge. Conversely, withholding levodopa can impair respiratory function, as the drug significantly improves parameters such as forced vital capacity, vital capacity, forced expiratory volume, and peak expiratory flow [14,15,16].

Infections exacerbate motor symptoms in PD, including bradykinesia, rigidity, and tremor [17,18]. Sepsis, coupled with gastrointestinal dysfunction, may destabilize levodopa absorption, leading to fluctuating blood concentrations that complicate extubation efforts and prolong mechanical ventilation dependence. Sudden levodopa withdrawal poses additional risks, including parkinsonism-hyperpyrexia syndrome, hyperthermia, and dysautonomia, which can lead to complications such as acute kidney failure, aspiration pneumonia, deep vein thrombosis/pulmonary embolism, and disseminated intravascular coagulation, further worsening sepsis outcomes [19]. These factors highlight the conflicting considerations in levodopa administration for PwP in septic shock, balancing the benefits to respiratory function against the increased need for inotropic support.

Given the controversies surrounding levodopa prescription in PwP with septic shock, this retrospective study utilized data from the Taipei Medical University Clinical Research Database (TMUCRD) to explore the complexities and challenges of levodopa administration in this critical scenario. By analyzing the medical records of PwP, the present study aimed to evaluate the therapeutic benefits and potential risks associated with levodopa use. The primary objective was to provide clinical guidance for managing levodopa treatment in PwP, particularly in the context of septic shock.

## 2. Methods

### 2.1. Institutional Review Board Approval

This study was approved by the Joint Institutional Review Board of Taipei Medical University (approval no. N202211031). Given the retrospective nature of this study, the requirement for informed consent was waived.

### 2.2. Data Source and Study Design

Data covering the period from 1 January 2004 to 31 December 2022 (updated in December 2023) were obtained from the TMUCRD. Generally, the TMUCRD collects comprehensive data from three major medical centers affiliated with Taipei Medical University in northern Taiwan: Taipei Medical University Hospital, Wan Fang Hospital, and Shuang Ho Hospital. These data include the electronic medical records of more than 4 million individuals, featuring both structured and unstructured information. In this study, to ensure confidentiality and privacy, all data were anonymized before being analyzed.

### 2.3. Participants

Patients meeting the following criteria were included in the study: being 45 years of age or older, having PD (International Classification of Diseases, Ninth Revision, Clinical Modification [ICD-9-CM] code 332.0 or International Classification of Diseases, Tenth Revision, Clinical Modification [ICD-10-CM] codes G20 and G21.4) in their medical records between 2004–2021, and having experienced septic shock (ICD-9-CM code 038 or ICD-10-CM codes A40, A41, and R65) requiring ICU admission. Patients meeting the following criteria were excluded from the analysis: having PD with a concurrent diagnosis of stroke (ICD-9-CM codes 430–438 or ICD-10-CM codes I60–I69), coronary artery disease (ICD-9-CM codes 410–414 and 429.2 or ICD-10-CM codes I20–I25), or cancer (ICD-9-CM codes 140–208 or ICD-10-CM codes C00–C96) during the same round of hospitalization. PwP were divided into exposure and nonexposure groups depending on their levodopa prescription claims (Anatomical Therapeutic Chemical code: N04BA01-03) during their septic shock period.

### 2.4. Study Outcomes

The outcomes of this study were the proportion and duration of inotrope prescription (Anatomical Therapeutic Chemical code: C01CA), the duration of mechanical ventilation dependence, and the 3-month mortality.

### 2.5. Statistical Analysis

Descriptive statistics were used to describe the characteristics of PwP who received levodopa (with levodopa group) and those who did not (without levodopa group). Chi-square tests were conducted to examine categorical variables, and Student’s *t* and Wilcoxon’s rank-sum tests were employed to examine parametric and nonparametric numerical variables. Multivariable linear regression models were used to evaluate the risk associated with an extended duration of inotrope prescription or length of mechanical ventilation dependence, with age, sex, comorbidities, and sepsis severity adjusted for. Adjusted hazard ratios (HRs) and Cox proportional hazards regression models were used to determine the differences in 3-month mortality between the two groups. All statistical analyses were conducted using Stata 15 software (StataCorp, College Station, TX, USA), with *p* values less than 0.05 considered statistically significant.

## 3. Results

**Figure 1:**
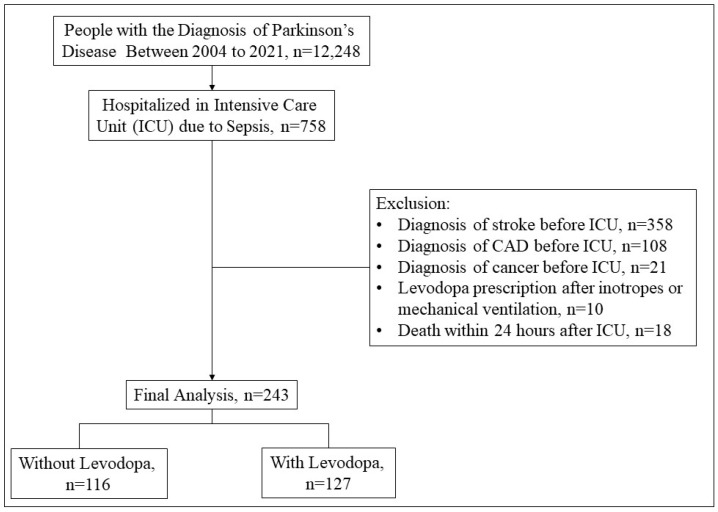
A flowchart of the patient selection process. CAD, coronary artery disease.

The selection process for this study began with identifying PwP between 2004 and 2021 (n = 12,248). From this cohort, patients who were hospitalized in the ICU due to sepsis were selected (n = 758). Exclusion criteria were then applied, which included the following: a diagnosis of stroke prior to ICU admission (n = 358), a diagnosis of coronary artery disease prior to ICU admission (n = 108), a diagnosis of cancer prior to ICU admission (n = 21), levodopa prescription initiated only after the administration of inotropes or mechanical ventilation (n = 10), and death within 24 h after ICU admission (n = 18). After applying these exclusions, a total of 243 patients were included in the final analysis. These individuals were subsequently divided into two groups based on levodopa prescription status during their ICU stay: those without levodopa (n = 116) and those with levodopa (n = 127) (Figure 1).

Among the selected PwP, no significant difference was discovered between the two groups in terms of age (without levodopa: 76.76 ± 10.81 years; with levodopa: 75.45 ± 8.53 years; *p* = 0.28) or sex distribution (without levodopa: 55 women; with levodopa: 62 women; *p* = 0.27). The prevalence of diabetes was higher in the without levodopa group than in the levodopa group (44.0% vs. 27.6%, *p* = 0.008), but the intergroup difference was nonsignificant in terms of the prevalence of hypertension (54.3% vs. 48.0%, *p* = 0.33), heart failure (25.0% vs. 26.8%, *p* = 0.75), cardiac arrhythmia (19.8% vs. 23.6%, *p* = 0.47), chronic kidney disease (24.1% vs. 14.5%, *p* = 0.07), and hepatic disease (14.7% vs. 10.2%, *p* = 0.30). Laboratory data revealed no significant differences between the two groups in terms of abnormal platelet count (13 out of 49 vs. 12 out of 72, *p* = 0.19), total bilirubin (16 out of 56 vs. 10 out of 68, *p* = 0.06), or c-reactive protein level (54 out of 58 vs. 79 out of 87, *p* = 0.76) (Table 1).

The incidence of mechanical ventilation (71 out of 5 vs. 77 out of 127, *p* = 0.93) and inotrope treatment (85 out of 116 vs. 92 out of 127, *p* = 0.88) was identical in the without/with levodopa groups (Table 2). Similarly, the median durations of mechanical ventilation dependence and inotrope treatment were identical in the two groups (Table 2). Multivariate logistic regression analysis—adjusted for age, comorbidities (diabetes, hypertension, heart failure, cardiac arrhythmia, chronic kidney disease, chronic hepatic disease, and chronic obstructive pulmonary disease), and laboratory tests (abnormal platelet count, total bilirubin and c-reactive protein)—revealed that treatment with levodopa did not significantly affect the duration of mechanical ventilation dependence (estimated: 6.37 days, 95% confidence interval [CI] = −3.63–16.37 days, *p* = 0.21), but it was associated with a longer duration of inotrope treatment (estimated: 3.43 days, 95% CI = 0.41–6.46 days, *p* = 0.027). Regarding the other variables, PwP with heart failure, chronic kidney disease, and an abnormal total bilirubin level experienced a significantly longer duration of inotrope dependence, but not mechanical ventilation dependence (Table 3). Comparing the PwP with/without an inotrope prescription also demonstrated that the proportions of heart failure, arrhythmia, and abnormal total bilirubin levels were significantly higher in PwP with an inotrope prescription (Table 4).

**Figure 2:**
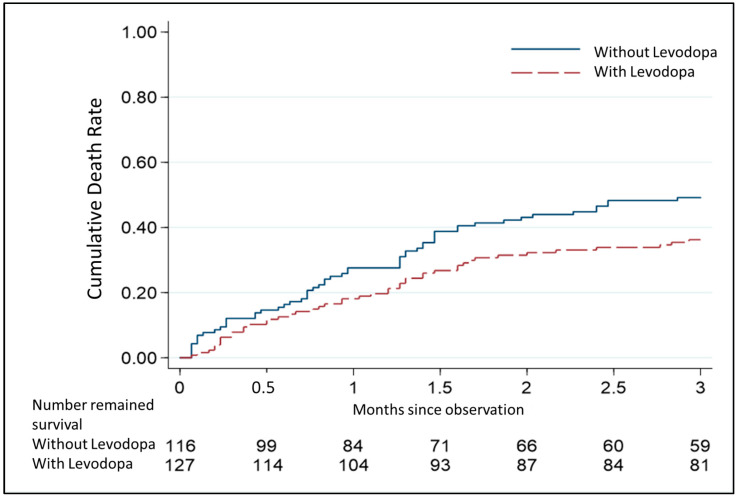
Cox proportional hazards regression curves of cumulative 3-month mortality in the with/without levodopa groups upon septic shock.

In PwP with septic shock, treatment with levodopa significantly reduced the risk of 3-month mortality (crude HR = 1.33, 95% CI = 1.01–2.22, *p* = 0.042). After age, sex, comorbidities (diabetes, hypertension, heart failure, cardiac arrhythmia, chronic kidney disease, chronic hepatic disease, and chronic obstructive pulmonary disease), and laboratory tests (abnormal platelet count, total bilirubin and c-reactive protein) were adjusted for, the results indicated that the adjusted HR indicated a trend, but this trend was nonsignificant (*p* = 0.068, Figure 2). Comparing the rates of survival and death in PwP during the 3-month follow-up period also demonstrated that age, proportion of heart failure, and abnormal total bilirubin level were significantly higher in the mortality group of PwP (Table 5).

## 4. Discussion

This study investigated the impact of levodopa administration on outcomes in PwP who experienced septic shock. It revealed that levodopa prescription is associated with a significantly longer duration of inotrope use, suggesting potential hemodynamic challenges. However, there was no significant difference in the duration of mechanical ventilation dependence between the levodopa and non-levodopa groups. Additionally, a nonsignificant trend toward reduced 3-month mortality among PwP who received levodopa was observed, highlighting its potential to influence survival outcomes in this critical care context. These results underscore the complex interplay between levodopa use and critical care interventions in PwP with septic shock.

Levodopa significantly affects cardiovascular function, particularly blood pressure regulation [14]. Studies have demonstrated that in PwP, the maximum tolerated doses of levodopa can induce a substantial reduction in erect systolic blood pressure without compensatory tachycardia [20,21]. This effect is attributed to levodopa’s enhancement of dopaminergic activity, which promotes vasodilation, reduces vascular resistance, and lowers blood pressure. This side effect is especially critical for PwP, who are already susceptible to blood pressure fluctuations due to the autonomic dysfunction associated with PD, increasing their risk of falls, weakness, and fatigue [22]. Beyond levodopa, other dopaminergic medications may also impact cardiovascular function and influence the effects of inotropes. Dopamine agonists, widely used as monotherapy or add-on therapy for PwP, are preferred due to their lower risk of dyskinesia and longer half-life. However, orthostatic hypotension is a common adverse effect [23]. Monoamine oxidase B inhibitors, which reduce dopamine degradation, enhance the therapeutic effect of levodopa. Nevertheless, selegiline has been found to diminish cardiovascular autonomic responses [24]. Therefore, careful monitoring of blood pressure is essential during levodopa and other dopaminergic therapies to mitigate complications related to hypotension.

During septic shock, systemic inflammation triggers profound changes in vascular function, including widespread vasodilation, increased vascular permeability, and reduced cardiac output [25]. These pathophysiological changes result in severe hypotension and impaired tissue perfusion, which are hallmark features of septic shock [26]. Inotropes play a vital role in stabilizing hemodynamics under these conditions by enhancing myocardial contractility to maintain adequate blood pressure and perfusion to vital organs [27]. However, levodopa may counteract the therapeutic effects of inotropes, potentially reducing their efficacy. Additionally, drug–drug interactions, such as those with linezolid, which is contraindicated with levodopa, can lead to unpredictable blood pressure fluctuations [28]. Gastrointestinal dysfunctions, including constipation and poor digestion, further complicate levodopa management by causing instability in its absorption and blood concentration, adding to the challenges of its use in septic shock patients. Furthermore, the present study identified an association between comorbidities—such as a medical history of heart failure and chronic kidney disease—and an extended period of inotrope requirement in PwP. These two health conditions are well-known substantial risk factors for prolonged ICU stays and inotrope dependence in people with septic shock, and our findings indicate that this association is also significant among PwP [29,30]. Although a history of chronic liver disease was not associated with the main outcomes of the present study, abnormal total bilirubin levels were linked to a higher incidence of inotrope prescription, prolonged dependence on inotropes, and an increased risk of 3-month mortality in PwP experiencing septic shock. Elevated bilirubin often indicates liver dysfunction in septic patients, potentially resulting from direct hepatocyte injury caused by bacterial products or a systemic inflammatory response. Its associations with sepsis severity and mortality are well documented [31,32]. In this study, including total bilirubin levels in the regression models helped us adjust for this potential confounding factor.

Septic shock often leads to respiratory complications, which may necessitate mechanical ventilation [33]. In PwP with septic shock, levodopa may be discontinued because of its effect on blood pressure. However, discontinuing levodopa can exacerbate the symptoms of PD, potentially affecting respiratory muscle function and prolonging the need for mechanical ventilation. This scenario is particularly concerning because PD itself can cause respiratory dysfunction [34,35]. The success of weaning from mechanical ventilation primarily depends on restoring adequate respiratory muscle function, which can be challenging in cases of ineffectively managed PD symptoms. Therefore, whether levodopa treatment should be continued in PwP experiencing septic shock remains a topic of debate, with no definitive evidence yet available to guide clinical decisions.

This study provides valuable insights into an underexplored yet clinically significant area—the impact of levodopa on septic shock management in PwP. Leveraging data from the TMUCRD, the study benefits from a comprehensive and reliable data source. Its use of robust statistical analyses, such as multivariable regression and Cox proportional hazards models, ensures a high degree of validity while adjusting for relevant confounders. The findings have direct clinical relevance, offering critical care specialists preliminary guidance on balancing the risks and benefits of levodopa use in septic shock. Additionally, the nuanced discussion of levodopa’s dual effects on motor function and hemodynamic stability provides a balanced perspective, emphasizing the complexity of clinical decision-making. The study also identifies key areas for future research, including the impact of Parkinson’s subtypes and disease stages on critical care outcomes, further enhancing its academic contribution. The potential advantages of continuous forms of levodopa administration, such as levodopa-carbidopa intestinal gel (LCIG), warrant further investigation in the context of septic shock. Unlike oral levodopa administration, LCIG provides a steady infusion that minimizes fluctuations in plasma drug concentrations, which could be particularly beneficial during septic shock when gastrointestinal dysfunction may impair drug absorption. By ensuring more stable dopaminergic stimulation, LCIG may reduce the risk of motor symptom exacerbation and facilitate better respiratory and autonomic function in PwP during critical care. Future studies should evaluate the feasibility, safety, and efficacy of LCIG in such scenarios to optimize management strategies for these high-risk patients.

In this study, the Hoehn and Yahr stage and exact motor dysfunction assessment results, such as the Unified Parkinson Disease Rating Score, were not available for every PwP. In PwP, admission to the ICU is not necessarily linked to their disease stage [36]. In the present study, it could not be determined whether patients were posture instability/gait difficulty (PIGD)-dominant or non-PIGD-dominant. PIGD-dominant patients are more likely to experience anti-PD-drug-induced hypotension compared with non-PIGD-dominant patients [37]. This factor may contribute to the outcomes observed in the ICU. As is the nature of healthcare database analysis, information about some novel factors of sepsis, such as orexin, could not be addressed in the present study. Some data support a hypothesis concerning the role of orexin in the fight-or-flight response; e.g., orexin administration causes an increase in cardiovascular and respiratory activity and analgesia [38]. In addition, this retrospective study has several limitations because it relies on a review of charts not initially created for research purposes, resulting in certain missing information, such as parameters like lung capacity, cough reflex, and airway resistance, which have been determined as factors of mechanical ventilation dependence. This study did not account for the potential interaction between levodopa and the anti-bacterial antibiotics used during septic shock. Certain antibiotics, particularly those altering gut microbiota, may impact the metabolism and absorption of levodopa, leading to fluctuations in plasma drug levels and possibly affecting its therapeutic efficacy. The exclusion of PwP with common comorbidities like stroke or coronary artery disease, while ensuring cohort homogeneity, restricts applicability to a broader PwP population. Additionally, the relatively small sample size may lack the statistical power needed to detect more nuanced differences during subgroup analyses, especially the effects of other dopaminergic medications, such as dopamine agonists and selegiline, which have been shown to exhibit a detrimental cardiovascular impact. Unmeasured confounding factors, such as nutritional status and ICU care quality, as well as the focus on short-term critical care outcomes rather than long-term recovery or quality of life, also limit the study’s scope. Finally, the misclassification of PD with other atypical degenerative parkinsonisms, particularly multiple system atrophy (MSA)—characterized by severe dysautonomia, poor levodopa response, and respiratory issues—represents an unavoidable limitation. Individuals with MSA were more likely to be classified into the non-levodopa group due to poor drug response, thereby diminishing the observed effect size of levodopa on prolonged inotrope dependence and mechanical ventilation requirements.

## 5. Conclusions

The present study underscores the complex nature of managing PwP in the ICU and emphasizes the importance of further research to identify the factors that affect the outcomes of these patients. Currently, no evidence-based recommendations are available in terms of how to taper or stop levodopa treatment in cases of septic shock, and most of the available data pertain to neuroleptic malignant syndrome. Therefore, it is recommended to refrain from abruptly discontinuing medications, although no official guidelines addressing this problem are yet available. With the population continuing to age, this problem is expected to become more prominent. Therefore, additional observational studies are urgently required.

## Figures and Tables

**Table 1:** The demographic information of the selected study subjects.

		Without Levodopa (n = 116)		With Levodopa (n = 127)		*p*-Value
			%		%	
Age (years old)		76.76 ± 10.81		75.45 ± 8.53		0.28
Female		52	44.8	66	52.0	0.27
Comorbidity						
	Hypertension	63	54.3	61	48.0	0.33
	Diabetes	51	44.0	35	27.6	0.008
	Heart Failure	29	25.0	34	26.8	0.75
	Arrhythmia	23	19.8	30	23.6	0.47
	CKD	28	24.1	19	14.5	0.07
	Chronic Liver Disease	17	14.7	13	10.2	0.30
	COPD	17	14.7	13	10.2	0.30
Abnormal Laboratory Data						
	Platelet	13/49		12/72		0.19
	Total Bilirubin	16/56		10/68		0.06
	CRP	54/58		79/87		0.76

CKD, chronic kidney disease; COPD, chronic obstructive pulmonary disease; CRP, C-reactive protein.

**Table 2:** The incidence and required treatment periods of inotropic agents and mechanical ventilation in people with Parkinson’s disease who experienced septic shock.

		Without Levodopa (n = 116)	With Levodopa (n = 127)	*p*-Value
Incidence				
	Inotropes	85	92	0.88
	Mechanical Ventilation	71	77	0.93
Treatment Period of Time (days) *				
	Inotropes	3 (2–11)	6.5 (2–15)	0.12
	Mechanical Ventilation	12 (5–30)	15 (8–25)	0.45

*, data were presented as median (Q1, Q3).

**Table 3:** The multiple-variable regression model analyzed the associations between the duration of each treatment (inotropes and mechanical ventilation) and all variables included in the study, such as levodopa prescription, comorbidities, and abnormal laboratory data, with adjustments for age and sex. The reference group consisted of individuals without a levodopa prescription (for levodopa prescription), without the according comorbidity (for each comorbidity), and with normal blood test results (for each blood test).

	Variable	Estimate	S.E.	95% CI	*p*-Value
Inotropes	Levodopa	3.43	1.54	0.41~6.46	0.027
	Hypertension	−3.33	1.59	−6.47~−0.20	0.04
	Diabetes	0.48	1.61	−2.69~3.65	0.77
	Heart Failure	6.50	1.80	2.95~10.05	<0.001
	Arrhythmia	1.01	1.82	−2.57~4.59	0.58
	CKD	5.30	1.96	1.44~9.16	0.007
	Chronic Liver Disease	−1.78	2.28	−6.27~2.71	0.43
	COPD	−1.56	2.31	−6.12~3.00	0.50
	Abnormal Platelet Count	4.40	2.72	−0.95~9.76	0.11
	Abnormal Total Bilirubin	6.05	2.63	0.83~11.23	0.02
	Abnormal CRP	3.54	3.49	−3.34~10.43	0.31
Mechanical Ventilation	Levodopa	6.37	5.07	−3.63~16.37	0.21
	Hypertension	3.45	5.25	−6.90~13.80	0.51
	Diabetes	−3.04	5.31	−13.52~7.43	0.57
	Heart Failure	9.87	5.96	−1.87~21.61	0.10
	Arrhythmia	0.61	6.01	−11.23~12.44	0.92
	CKD	9.90	6.46	−2.84~22.64	0.13
	Chronic Liver Disease	−5.37	7.53	−20.20~9.47	0.476
	COPD	13.43	7.64	−1.63~28.50	0.08
	Abnormal Platelet Count	−1.87	8.98	−19.57~15.84	0.84
	Abnormal Total Bilirubin	1.50	8.69	−15.62~18.62	0.86
	Abnormal CRP	8.70	11.55	−14.05~31.46	0.45

CI, confidence interval; CKD, chronic kidney disease; COPD, chronic obstructive pulmonary disease; CRP, C-reactive protein; S.E., standard error.

**Table 4:** A comparison of the demographic information of the study subjects with/without inotropes.

		Without Inotropes (n = 66)		With Inotropes (n = 177)		*p*-Value
			%		%	
Age (years old)		75.39 ± 9.27		76.33 ± 9.61		0.50
Female		29	43.9	96	54.24	0.15
Comorbidity						
	Hypertension	36	54.55	88	49.72	0.503
	Diabetes	22	33.33	64	36.16	0.682
	Heart Failure	7	10.61	56	31.64	0.001
	Arrhythmia	6	9.09	47	26.55	0.003
	CKD	10	15.15	37	20.90	0.313
	Chronic Liver Disease	4	6.06	26	14.69	0.069
	COPD	5	7.58	25	14.12	0.168
Abnormal Laboratory Data						
	Platelet	7/44		18/80		0.49
	Total Bilirubin	2/28		24/96		0.04
	CRP	34/40		99/105		0.09

CKD, chronic kidney disease; COPD, chronic obstructive pulmonary disease; CRP, C-reactive protein.

**Table 5:** A comparison of the demographic information of the study subjects and their survival or death during the 3-month follow-up period.

		Survival (n = 140)		Death (n = 103)		*p*-Value
			%		%	
Age (years old)		74.92 ± 9.44		77.64 ± 9.42		0.03
Female		68	48.6	57	55.34	0.30
Comorbidity						
	Hypertension	74	52.86	50	48.54	0.506
	Diabetes	54	38.57	32	31.07	0.227
	Heart Failure	26	18.57	37	35.92	0.002
	Arrhythmia	27	19.29	26	25.24	0.267
	CKD	25	17.86	22	21.36	0.495
	Chronic Liver Disease	13	9.29	17	16.50	0.091
	COPD	14	10.00	16	15.53	0.195
Abnormal Laboratory Data						
	Platelet	16/76		9/45		0.89
	Total Bilirubin	10/69		16/55		0.047
	CRP	80/86		53/59		0.49

CKD, chronic kidney disease; COPD, chronic obstructive pulmonary disease; CRP, C-reactive protein.

## Data Availability

Please contact the corresponding authors (W.T. Chiu and L. Chan). Accessing the data and materials requires permission from the TMU-JIRB.
